# The effects of progressive resistance training combined with a whey-protein drink and vitamin D supplementation on glycaemic control, body composition and cardiometabolic risk factors in older adults with type 2 diabetes: study protocol for a randomized controlled trial

**DOI:** 10.1186/1745-6215-15-431

**Published:** 2014-11-06

**Authors:** Robin M Daly, Eliza G Miller, David W Dunstan, Deborah A Kerr, Vicky Solah, David Menzies, Caryl A Nowson

**Affiliations:** Centre for Physical Activity and Nutrition Research, Deakin University, Melbourne, VIC Australia; Baker IDI Heart and Diabetes Institute, Melbourne, VIC Australia; School of Public Health, Curtin University, Perth, WA Australia; Fitness Australia, Alexandria, NSW Australia

**Keywords:** Older adults, Progressive resistance training, Randomized controlled trial, Study protocol, Type 2 diabetes, Vitamin D, Whey-protein

## Abstract

**Background:**

While physical activity, energy restriction and weight loss are the cornerstone of type 2 diabetes management, less emphasis is placed on optimizing skeletal muscle mass. As muscle is the largest mass of insulin-sensitive tissue and the predominant reservoir for glucose disposal, there is a need to develop safe and effective evidence-based, lifestyle management strategies that optimize muscle mass as well as improve glycaemic control and cardiometabolic risk factors in people with this disease, particularly older adults who experience accelerated muscle loss.

**Methods/Design:**

Using a two-arm randomized controlled trial, this 6-month study builds upon the community-based progressive resistance training (PRT) programme *Lift for Life®* to evaluate whether ingestion of a whey-protein drink combined with vitamin D supplementation can enhance the effects of PRT on glycaemic control, body composition and cardiometabolic health in older adults with type 2 diabetes. Approximately 200 adults aged 50 to 75 years with type 2 diabetes, treated with either diet alone or oral hypoglycaemic agents (not insulin), will be recruited. All participants will be asked to participate in a structured, supervised PRT programme based on the *Lift for Life®* programme structure, and randomly allocated to receive a whey-protein drink (20 g daily of whey-protein plus 20 g after each PRT session) plus vitamin D supplements (2000 IU/day), or no additional powder and supplements. The primary outcome measures to be collected at baseline, 3 and 6 months will be glycated haemoglobin (HbA_1c_) and insulin sensitivity (homeostatic model assessment). Secondary outcomes will include changes in: muscle mass, size and intramuscular fat; fat mass; muscle strength and function; blood pressure; levels of lipids, adipokines and inflammatory markers, serum insulin-like growth factor-1 and 25-hydroxyvitamin D; renal function; diabetes medication; health-related quality of life, and cognitive function.

**Discussion:**

The findings from this study will provide new evidence on whether increased dietary protein achieved through the ingestion of a whey-protein drink combined with vitamin D supplementation can enhance the effects of PRT on glycaemic control, muscle mass and size, and cardiometabolic risk factors in older adults with type 2 diabetes.

**Trial registration:**

Australian New Zealand Clinical Trials ACTRN12613000592741.

**Electronic supplementary material:**

The online version of this article (doi:10.1186/1745-6215-15-431) contains supplementary material, which is available to authorized users.

## Background

Type 2 diabetes is one of the most prevalent chronic metabolic diseases of the twenty-first century [1, 2]. In 2011, there were 366 million people with diabetes globally, a figure projected to rise to 552 million by 2030 [3]. The human and economic burden of illness associated with type 2 diabetes contributes significantly to ill health, disability and premature death, and is further exacerbated by the onset of micro- and macrovascular complications. Thus, there is a need to develop safe, effective and sustainable population-based prevention and management strategies that collectively improve multiple risk factors associated with this disease.

Lifestyle modification combining energy restriction, weight loss and physical activity remains the cornerstone of type 2 diabetes prevention and treatment [4, 5]. While a reduction in body weight and improvements in glycaemic control have been observed following energy restriction and aerobic or endurance activity, a concomitant loss in muscle mass can also occur [6, 7]. Skeletal muscle is critical for people with type 2 diabetes, as it is the largest mass of insulin-sensitive tissue and the predominant reservoir for glucose disposal. Losses in muscle mass can negatively impact metabolic rate, compound the problems of insulin resistance and lead to reduced physical function and quality of life [8, 9]. As a result, current international consensus exercise guidelines recommend that progressive resistance training (PRT) be incorporated into the overall physical activity plan for people with type 2 diabetes, owing to its positive effects on muscle mass and glycaemic control [5]. Indeed, our previous research has demonstrated that high-intensity PRT is safe and effective for improving glycated haemoglobin (HbA_1c_) levels and lean tissue mass in older adults with type 2 diabetes [10–12]. This work led to the development of a national community-based PRT programme, entitled *Lift for Life®*[13]*,* with the goal of providing greater access for adults with, or at risk, of type 2 diabetes, to participate in an evidence-based PRT programme within existing community health and fitness centres through the development of a network of accredited providers [14].

Since nutritional management is also an important component in the treatment of type 2 diabetes, combining PRT with dietary modification may offer a synergistic and incremental effect on glycaemic control as well as body composition and cardiometabolic risk factors. While the optimal macronutrient composition of the diet for the management of type 2 diabetes remains uncertain, emerging evidence suggests that there are health benefits associated with high-protein diets in overweight and obese adults and those with type 2 diabetes. For instance, a meta-analysis of nine randomized controlled trials ranging from 4 to 24 weeks reported that high-protein diets had beneficial effects on weight loss and HbA_1c_ levels and tended to reduce blood pressure in people with type 2 diabetes, with no adverse effects on blood lipids [15]. Despite these positive findings, dietary studies controlling for macronutrient composition are often difficult to implement in the ‘real-world’, as they require an individual to follow a prescribed meal plan, and adherence often relies on self-reported dietary intake. Therefore, the addition of a protein supplement might be a more practical and effective approach, as it does not require individuals to make marked changes in their usual dietary habits. This approach was used in a study of overweight and obese adults and showed that a daily intake of greater than 30% energy from protein achieved through a whey-protein supplement, compared with a diet containing approximately 16% of energy from protein through habitual intake, was more effective for improving lipid levels and insulin sensitivity [16]. Importantly, energy intakes remained similar between the groups throughout the study, and there was no effect of the intervention on body composition. A number of other short-term studies have also reported that whey-protein has insulinotropic properties that can improve insulin sensitivity and glycaemic control in people with type 2 diabetes [17–19].

In non-diabetic adults, there is compelling evidence that ingestion of whey-protein soon after exercise can augment the anabolic benefits of PRT on muscle mass [20, 21]. While there is still ongoing research into defining whether there is an optimal dose of protein needed to elicit a synergistic skeletal muscle response with PRT, particularly in older adults, several recent studies and reviews have recommended that 20 to 40 g of high-quality, rapidly digested, leucine-rich protein, such as whey-protein, be consumed soon after each bout of PRT to maximally stimulate muscle protein synthesis and promote muscle hypertrophy [22–24]. Given that skeletal muscle is the primary site of glucose disposal, and is highly responsive to exercise as a stimulus to increase glucose uptake, we hypothesize that combining PRT with whey-protein might represent an optimal strategy to enhance muscle hypertrophy and glycaemic control, and improve cardiometabolic risk factors in people with type 2 diabetes.

There are also other lifestyle factors that might have beneficial effects on skeletal muscle. For instance, there is mounting evidence that treatment with vitamin D can have positive effects on muscle, including strength and function [25, 26]. Low serum 25-hydroxyvitamin D (25(OH)D) levels might also play a role in the development of type 2 diabetes [27, 28], with reports that vitamin D deficiency is associated with impaired β-cell function, glucose intolerance and insulin resistance [28]. Although the findings from randomized controlled trials examining the effects of supplemental vitamin D, alone or with calcium, on measures of glycaemic control, insulin sensitivity and secretion have been inconclusive, there is some evidence for a beneficial effect in adults with or at increased risk of type 2 diabetes [29–32]. There are also reports that vitamin D might have anti-inflammatory properties, particularly in people with various pathological conditions, such as type 2 diabetes [33, 34]. This is important because chronic low-grade inflammation has been linked to accelerated muscle loss and has emerged as the common denominator linking type 2 diabetes, metabolic syndrome, insulin resistance, endothelial dysfunction and cardiovascular disease [28]. While the optimal serum 25(OH)D concentration for muscle and health benefits remains hotly debated, we hypothesize that vitamin D treatment combined with whey-protein supplementation and PRT will be more effective for improving glycaemic control, body composition and various inflammatory and cardiovascular risk factors in people with type 2 diabetes than PRT alone.

The primary aim of this randomized controlled trial is to examine whether a community-based PRT programme combined with additional whey-protein and vitamin D can promote greater improvements in glycaemic control and insulin sensitivity than PRT alone in older adults with type 2 diabetes. Secondary aims of the study are to assess the effects of the intervention on changes in: (1) total body and regional lean tissue mass and fat mass, thigh muscle cross-sectional area and muscle density (as a surrogate measure of intramuscular fat infiltration), muscle strength and functional performance; (2) cardiovascular risk factors, including blood pressure and blood lipid levels, (3) levels of adipokines and inflammatory markers, and (4) quality of life and cognitive function. In addition, we will examine whether changes in lean tissue mass, muscle size, density and strength and a reduction in metabolic or inflammatory markers are predictive of any exercise-induced improvements in glycaemic control and insulin sensitivity.

## Methods/Design

### Study design

This study is a 6-month, two-arm, parallel, randomized controlled trial. Participants with confirmed type 2 diabetes will be asked to participate in a structured PRT programme, based on the community-based *Lift for Life®* programme, and randomly allocated to receive either a whey-protein powdered drink plus vitamin D supplements, or no additional powder or supplements. A matched placebo powder was deemed inappropriate for this trial, as the use of food additives, such as maltodextrin, has the potential to influence the outcome measures (for example, glycaemic control). The selection of a two-arm design (for example, the lack of a true ‘non-exercise’ control arm) is consistent with our primary research aim, which seeks to examine whether whey-protein plus vitamin D can enhance the health benefits of PRT in older adults with type 2 diabetes. The trial is managed by the Centre for Physical Activity and Nutrition Research at Deakin University, Burwood, Melbourne, Victoria, Australia and is funded by a National Health and Medical Research Council Project Grant (ID1046269). The study has been approved by the Deakin University Human Research Ethics Committee (HREC 2013–050), and is registered with the Australian and New Zealand Clinical Trials Registry (ACTRN12613000592741).

### Participants

Men and women aged 50 to 75 years with established type 2 diabetes, treated with diet alone or any oral hypoglycaemic agents (except insulin), will be invited to participate in the study.

#### Recruitment

Participants living in the Melbourne metropolitan and surrounding areas in Victoria, Australia with type 2 diabetes will be recruited into the study via state and local media campaigns, including newspaper and radio advertisements, flyers, web-based media and word of mouth. This will be further supplemented with letters sent to local doctors, endocrinologists, pharmacists, diabetic educators and support groups, asking them to place advertisements in their facilities and inviting them to refer patients with type 2 diabetes to the study. A letter will also be sent to participants with type 2 diabetes who are registered on the National Diabetes Services Scheme, which is an initiative of the Australian Government administered by Diabetes Australia, to participate in the trial. All participants who express an interest in the study will undergo screening to determine their eligibility to participate in the trial based on the outlined criteria.

#### Screening and eligibility

Eligibility for the study will be based on a two-step screening process. First, all participants will be screened via a telephone questionnaire and ineligibility will be based on the following criteria: (1) HbA_1c_ >10%; (2) current or prior participation in a structured PRT programme >1 session per week or moderate-intensity physical activity ≥150 min/week in the previous 3 months; (3) vitamin D or calcium supplement use >500 IU/day and >600 mg/day, respectively, in the previous 3 months; (4) severe orthopaedic, cardiovascular or respiratory conditions that would preclude participation in an exercise programme, or those with absolute contraindications to exercise, according to American College of Sports Medicine guidelines [35]; (5) renal impairment (eGFR <45 ml/(min 1.73 m^2^)) or disease; (6) regular use of protein supplements; (7) conditions that may affect vitamin D or calcium metabolism; (8) current smoker, or (9) body mass index >40 kg/m^2^. Participants will be encouraged and monitored to keep constant throughout the study any use of lipid-lowering or anti-hypertensive medication and not modify their lifestyle habits other than necessary for the study. To increase the external validity of the study and because of a lack of consensus with regard to the optimal serum 25(OH)D concentration for health benefits, vitamin D status will not be an inclusion or exclusion criterion but will be assessed as part of the biochemical analysis. Data from the 1999 to 2000 Australian Diabetes, Obesity and Lifestyle (AusDiab) study, involving more than 11,000 Australians, indicates that 89% of adults with type 2 diabetes had insufficient serum 25(OH)D levels (<75 nmol/l) and 55% were vitamin D deficient (<50 nmol/l) (RMD, unpublished observation). Participants who pass the initial telephone screening will be required to obtain approval from their local doctor to clear them of any contraindicated medical conditions to exercise, based on American College of Sports Medicine guidelines, to participate in the programme. Participants will also be asked to provide a fasted, morning blood sample to confirm that their HbA_1c_ level is <10%. Written informed consent will be obtained from all participants prior to commencing the programme.

#### Randomization and blinding

**Figure 1:**
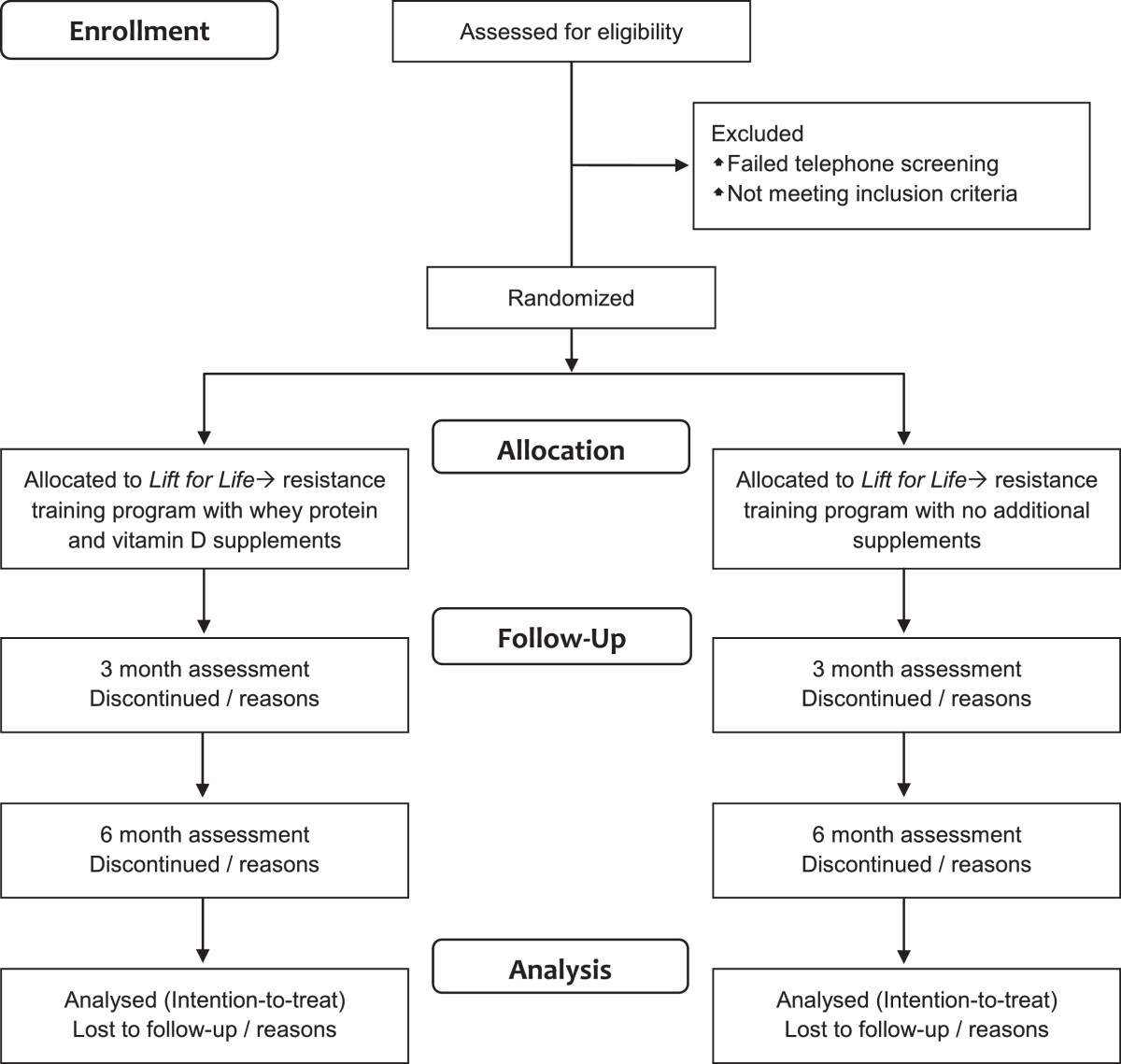
**Flow diagram of progress from screening to the final follow-up assessment.**

Following completion of baseline testing, participants will be randomized, stratified by sex and diabetes treatment (diet or oral hypoglycaemic agents), in blocks of four using a computer-generated random number sequence by an independent researcher. All research staff involved in the assessments will be blinded to the group allocation. The programme coordinator will be solely responsible for the distribution of the protein powder and vitamin D supplements. A flow diagram of the study protocol is outlined in Figure 1.

### Intervention

#### Progressive resistance training programme

All participants involved in the study will be asked to complete 24 weeks of resistance training (64 sessions in total), based on the successful *Lift for Life®* PRT programme, in community-based health and fitness centres. The *Lift for Life®* programme is a structured, evidence-based PRT programme that is designed for people with, or at risk, of developing type 2 diabetes. It has been developed from a series of previous laboratory and community-based exercise intervention studies demonstrating that PRT is safe and effective for improving glycaemic control and body composition in people with type 2 diabetes [10–12]. The programme will consist of moderate to high-intensity PRT (three sets of eight to ten repetitions at a weight that cannot be lifted for more than eight to ten repetitions) involving dynamic concentric and eccentric contractions targeting all the major muscle groups and with an emphasis on weekly progressive overload (increments of 2 to 10%). Participants will train twice a week for the first 8 weeks, and three times weekly for the remainder of the programme. Training sessions will last 45 to 60 minutes and will be supervised by qualified trainers (accredited exercise physiologists, physiotherapists and experienced Certificate IV personal trainers). All participants enrolled in the study will be charged a fee by each local provider to undertake the 24-week programme, at a cost of approximately Australian $5 to $13 per session, but will be eligible for reimbursement up to the value of Australian $240 at the completion of the study based on their level of exercise compliance (for example, 80% compliance will equate to a $192 rebate).

The 24-week *Lift for Life®* PRT programme is divided into three phases (bronze, silver, gold), each 8 weeks in duration. During the initial phase (bronze), participants receive education on the benefits of the programme, safety and correct exercise techniques and are asked to attend two supervised exercise sessions per week. After 8 weeks, all programmes will be reviewed and a new customized programme will be designed for each participant (silver), but with the aim of participating in one additional unsupervised exercise session to achieve the goal of three PRT sessions per week. The final 8-week phase (gold) is a replication of the previous phase with a new customized and progressively challenging programme. Throughout the programme, all completed exercises, sets and repetitions for each session will be recorded on *Lift for Life®* exercise cards, to monitor progression and compliance. Further details of the programme, including examples of the exercises and intensity prescribed, have been reported previously [14].

One of the challenges when implementing exercise programmes in community settings is that of enhancing adoption and adherence to these programmes. At the foundation of *Lift for Life®* and central to its success are individually tailored PRT programmes and supervised training sessions. The supervised sessions serve two purposes. First, the programme aims to emulate the level of attention received by participants in the previous trials on which the exercise guidelines and *Lift for Life®* programme are based. Second, previous research demonstrates that unsupervised training is less effective in translating to health benefits [12, 36]. Exercise programmes prescribed as part of the *Lift for Life®* programme are individually tailored, so comorbidities or complications (for example, knee osteoarthritis) can be taken into consideration with exercises appropriately modified on a case-by-case basis. Further, to enhance compliance and retention, all participants will receive monthly follow-up phone calls, and will be eligible to receive a reimbursement (as indicated previously) to cover the costs associated with their participation in the programme.

#### Whey-protein drink and vitamin D supplementation

Participants randomized to the protein and vitamin D group will receive a 3-month supply of a whey-protein enriched powder and vitamin D supplements at their baseline testing appointment and again after 12 weeks. The protein powder will be provided in a single-use sachet form (OmniBlend, Campbellfield, Victoria, Australia). Participants will be instructed to add 150 ml of cold water to a calibrated ‘shake-and-take’ container and then add one sachet (powder) containing 20 g of whey-protein concentrate 80%, containing approximately 2.4 g of leucine, to the water, close the lid and shake vigorously for at least ten shakes and consume immediately. Participants will be instructed to consume one drink every morning before breakfast and one drink within two hours of each PRT session. Thus, they will consume a total of seven drinks per week, with an extra drink on each of the two training days during the first 8 weeks. This will increase to ten drinks per week when participants move to three sessions per week. This will raise the total supplemental protein dose from 20 g on non-training days to 40 g on training days. Each protein drink will provide approximately 335 kJ of energy, 2 g of lactose, 1 g of fat and 5 g of fibre. Participants will also be asked to take two 1000 IU capsules of Vitamin D_3_ (Ostelin, Melbourne, Victoria, Australia) every evening for the duration of the study. The goal of vitamin D treatment is to raise serum 25(OH)D concentrations to at least 75 nmol/l. All supplements and the protein powder will be provided free of charge.

### Outcome measures

A summary of the proposed outcome measures is shown in Table 1. Participants will attend Deakin University twice throughout the duration of the study for assessment (at baseline and after 24 weeks), with an intermediary assessment of anthropometry, muscle strength, physical and cognitive function and repeat questionnaires conducted on-site at each health and fitness centre after 12 weeks. With the exception of the fasted blood sample collection, which will occur at local pathology clinics at baseline, 12 and 24 weeks, and the intermediary assessment, all other outcome measures will be assessed within the School of Exercise and Nutrition Sciences at Deakin University, Burwood, Melbourne, Victoria, Australia.

**Table 1:** **Summary of the outcome measures**

Variables	Data collection method	Data collection points		
		Baseline	3 months	6 months
**Primary outcome measures**				
Glycated haemoglobin (HbA_1c_)	Overnight, fasted serum sample	×	×	×
HOMA-2 insulin resistance	Overnight, fasted serum sample, HOMA-2 calculator	×	×	×
HOMA-2 β-cell function	Overnight, fasted serum sample, HOMA-2 calculator	×	×	×
**Secondary outcome measures**				
Body composition	Dual energy X-ray absorptiometry total body and regional lean mass and fat mass, and bone density	×		×
	Peripheral quantitative computed tomography scan at 25% femur site	×		×
Biochemistry and hormonal measures	Routine biochemistry	×	×	×
	Serum 25-hydroxyvitamin D	×	×	×
Adipokines and inflammatory markers	Overnight fasted plasma collection	×	×	×
Blood lipids	Overnight fasted plasma collection	×	×	×
Blood pressure	Automated measurement	×	×	×
Muscle strength	Leg press and seated row	×		×
	Isometric knee extensor strength	×	×	×
Muscle function and balance	Timed up-and-go test	×	×	×
	Four-square step test	×	×	×
	30-second sit-to-stand test	×	×	×
Diabetes and other medication	Monthly reports	×	×	×
Health-related quality of life	Short Form (36) version 2 questionnaire	×	×	×
Cognitive function	CogState Brief Battery computerized tests	×	×	×
**Additional measures**				
Anthropometry	Height, weight, body mass index and waist circumference	×	×	×
Physical activity	Community Healthy Activities Model Program for Seniors questionnaire	×	×	×
Diet	Anti-Cancer Council Food Frequency questionnaire	×		×
	24-hour food recollection	×	×	×
Adverse events	Monthly phone calls	Collected from monthly calls		
Programme adherence	Calculated from monthly calendars collected every 8 weeks	Collected every 8 weeks		
Supplement compliance	Calculated from supplement and sachets returned at 3 and 6 months		×	×

HOMA, homeostasis model assessment.

#### Primary outcome measures

The primary outcome measures will be changes in glycated haemoglobin levels (HbA_1c_) and homeostasis model assessment 2 (HOMA-2) of insulin resistance and β-cell function based on model-derived estimates using the validated HOMA-2-calculator [37], version 2.2.3, from fasting glucose and insulin measured at least three days after the last PRT session. All data will be checked prior to importing into the calculator, and extreme values of fasting plasma glucose ≤3 or ≥25 mmol/l or serum insulin <20 or >300 pmol/l will be excluded, as this is the validity range of the HOMA-2 calculation, based on specific insulin measures. More specific details of the blood collection and methodology for assessing these measures are provided later.

#### Secondary outcome measures

Secondary outcome measures will include changes in: body composition (lean tissue mass, muscle size and intramuscular fat), fat mass, muscle strength, blood pressure, blood lipids, adipokines and inflammatory markers, serum insulin-like growth factor-1 and 25(OH)D, renal function, diabetes medication (purpose, variety and dosage), health-related quality of life and cognitive function. Other covariates and variables of interest to be assessed include: anthropometry, habitual physical activity, diet and use of lipid-lowering and blood pressure medication. A summary of all the outcome measures is shown in Table 1.

##### Body composition

Total body and regional (arms and legs) lean tissue mass, fat mass and percentage body fat will be assessed using dual energy X-ray absorptiometry (Lunar Prodigy, GE Lunar Corp., Madison, WI, USA), using software version 12.30.008. A peripheral quantitative computed tomography scanner (XCT 3000, Stratec Medizintechnik GmbH, Pforzheim, Germany) will be used to measure muscle cross-sectional area, subcutaneous fat cross-sectional area and muscle density, as a surrogate measure of intermuscular adiposity, at the 25% femur site using methods previously reported [38]. Briefly, after performing a scout view of the distal end of the femur, scans will be taken at the 4% and 25% position of the femur. The slice thickness will be 2.3 mm, and the voxel size will be set at 0.3 mm at a scanning speed of 10 mm/s. Subcutaneous fat cross-sectional area will be determined by selecting the area with thresholds -40 to +40 mg/cm^3^ hydroxyapatite density (contour mode 3, peel mode 1), and the muscle cross-sectional area will be determined by subtracting the total bone cross-sectional area (threshold, 280 mg/cm^3^; contour mode 1, peel mode 2) and subcutaneous fat cross-sectional area from the total area of the distal femur (threshold, -40 mg/cm^3^, contour mode 3, peel mode 1). For consistency, all analysis of dual energy X-ray absorptiometry and peripheral quantitative computed tomography will be undertaken by a single investigator. The short-term coefficient of variation for repeated measurements of total body lean mass and fat mass in our laboratory ranges from 1.0% to 1.7%. The coefficient of variation for the femur muscle cross-sectional area is 1.3%.

##### Biochemical, hormonal and inflammatory markers

Following an overnight fast, participants will attend one commercial pathology clinic with multiple collection centres where rested, morning (8 to 10 am) venous blood samples will be collected. All blood will be sent to a central pathology laboratory accredited by the National Association of Testing Authorities Royal College of Pathologists Australasia. HbA_1c_ will be assessed by cation exchange HPLC using a Bio-Rad VARIANT II turbo HbA_1c_ kit-2.0 (Bio-Rad Laboratories, Hercules, CA, USA). Fasting plasma glucose will be assessed using the hexokinase method (Roche Diagnostics, Mannheim, Germany). Levels of C-peptide will be assessed using an electrochemiluminescence immunoassay and of high sensitivity C-reactive protein by an Immunoturbidimetric assay from Roche Diagnostics (Mannheim, Germany). Total cholesterol, high-density lipoprotein cholesterol and triglycerides will be determined using an enzymatic colorimetric method (Roche Diagnostics, Mannheim, Germany). Low-density lipoprotein cholesterol will be calculated using Friedewald’s formula. Serum creatinine, urea, albumin, calcium and phosphorus levels will be analyzed using standardized techniques. The estimated glomerular filtration rate (eGFR), as a measure of kidney function, will be calculated using the participants’ serum creatinine, age and sex according to the abbreviated ‘modification of diet in renal disease’ formula, which is now used by most laboratories in Australia:

eGFR (ml/(min 1.73 m^2^) = 175 × [serum creatinine (μmol/l) × 0.0113]^–1.154^ × age (years)^-0.203^] for men and eGFR (ml/(min 1.73 m^2^) = 175 × [serum creatinine (μmol/l) × 0.0113]^–1.154^ × age (years)^-0.203^ × 0.742 for women.

Serum aliquots will also be collected and stored at -80°C so that the following parameters can be assessed in a single batch at the completion of the study: levels of serum insulin, serum 25(OH)D, serum insulin-like growth factor 1, serum adiponectin and resistin, and a battery of pro-inflammatory and anti-inflammatory cytokines, including IL-6, IL-1β, IL-8, TNF-α and IL-10.

##### Blood pressure

After a 5-minute rest period seated in a quiet room, systolic and diastolic blood pressure will be measured using an automated blood pressure monitor (A&D Instruments, Oxon, UK). Four measurements will be taken on the left arm with a 2-minute interval between readings; the mean of the final three readings will be used in the analysis.

##### Muscle strength

Muscle strength of the lower limbs and upper back will be measured by employing a three-repetition maximum strength test for leg press and seated row exercises. This test determines the heaviest weight that can be used to complete three complete repetitions of an exercise whilst maintaining correct form and technique, and corresponds to ≈ 85% of an individual’s one-repetition maximum strength. Prior to the three-repetition maximum muscle strength test, participants will complete a 5-minute warm-up on an exercise bike. To determine the three-repetition maximum strength, each participant will initially perform a warm-up set of eight to ten repetitions with a light load. After successful completion of a further six to eight repetitions at a heavier weight selected by the instructing researcher and following a brief rest (≈2 to 3 minutes), the workload will be increased incrementally until only three repetitions with correct technique can be completed. For each participant, the formula employed by Wathen *et al.*[39] will be used to calculate each participant’s leg and back one-repetition maximum strength. In addition, isometric knee extensor strength will be measured on the participant’s dominant leg using Lord’s strap assembly, incorporating a strain gauge (Neuroscience Research Australia, Sydney, New South Wales, Australia). Participants will have one practice trial followed by two maximal tests with a 60 second rest between each test. This test has been shown to have excellent test-retest reliability (Pearson’s *r* = 0.92) [40]. For analysis, knee extension strength will be expressed per unit of lower leg length to compensate for the length of the lever arm.

##### Physical function

The timed up-and-go test, four-square step test and 30-second sit-to-stand test will be used to assess muscle function.

The timed up-and-go test is a measure of dynamic balance during three commonly performed functional activities: standing up from and sitting down in a chair, walking, and turning [41]. Briefly, participants will be seated in a chair (height 45 cm) that will be placed at the end of a marked 3 m walkway. On the command ‘go’, participants will be instructed to stand up, walk at a comfortable speed for 3 m, turn, walk back to the chair and sit down. To minimize any ceiling effects and make the test more challenging, the participants will also be instructed to start counting backwards in threes from a random number. All participants will be given a practice trial and one test run. A stopwatch will be used to record the time taken (in seconds) to complete the test. This test has an established interrater reliability, with an intraclass correlation of 0.99 [42].

The four-square step test is a clinical test used to assess dynamic standing balance and stepping speed in four different directions [43]. This test has been shown to have high interrater (intraclass correlation, 0.99) and retest reliability (intraclass correlation, 0.98) [43]. To complete this test, participants will be required to step forwards, sideways and backwards over four canes resting flat on the floor in a cross formation, moving first in a clockwise and then in a counterclockwise direction to return to the starting position. Participants will be instructed to complete the task as quickly as possible without touching or stepping on the canes, and if possible, to face forwards during the entire sequence. They will also be instructed to ensure that both feet make contact with the floor in each square. After one practice trial, participants will complete the test and the time (in seconds) taken to complete the sequence will be measured with a stopwatch and recorded as the final score.

The 30-second sit-to-stand test provides a measure of lower-extremity muscle strength and function and is administered in a chair without arms [44]. Participants start from a seated position in the chair, with arms folded across the chest, and are instructed to stand fully upright and then return to the seated position at their own pace as many times as possible in 30 seconds. The final score will be the number of complete stands recorded during this time. This test has been shown to have good reliability with a test-retest intraclass correlation of 0.84 to 0.92 in a community-dwelling sample of older men and women aged 60 years and over [44].

##### Dietary habits

The Anti-Cancer Council Food Frequency questionnaire will be used to quantify habitual eating habits and provide data on macro- and micronutrient intake [45]. Dietary data collected from the Anti-Cancer Council Food Frequency questionnaire will be supplemented with 24-hour dietary recollections completed at baseline, 12 and 24 weeks. Participants will be contacted (via phone) by the research staff (trained in completing food recall tasks) and asked to report all food and drink consumed over the previous 24-hour period. A standard script will be used and the ‘triple pass’ method will be utilized to maximize the ability of respondents to recall what was consumed. Household measures (measuring cups, plates, bowls and glasses) will be used to help estimate food portion sizes and participants will be provided with a standard portion size booklet. The data collected from the 24-hour recalls will be entered and analyzed using Australia-specific dietary analysis software (FoodWorks, Xyris software, Highgate Hill, Queensland, Australia).

##### Anthropometry

Height will be measured to the nearest 0.1 cm with a wall-mounted stadiometer and body weight to the nearest 0.1 kg using calibrated electronic digital scales. Waist circumference will be measured on a horizontal plane, 2 cm proximal to the uppermost lateral border of the right iliac crest.

##### Physical activity

Total leisure and recreational physical activity time (hours per week) will be assessed using the Community Healthy Activities Model Program for Seniors physical activity questionnaire. This questionnaire has been specifically designed for use in older adults and found to be reliable, valid and sensitive to change [46]. Participants will document the frequency and duration of their participation in a ‘typical week’ of the preceding four weeks. The results will be reported as estimated kilojoules per week spent in moderate to high-intensity activities.

##### Health and medical history and medication use

All participants will complete a lifestyle questionnaire to obtain information on education background, current and previous employment details, history of diseases or illnesses, family history of diabetes, smoking history, current medication and dietary supplement use, average weekly alcohol consumption, weekly television viewing and sitting time, and sun exposure habits. For women, menstrual history, including age of onset of menopause and menstrual cycle regularity, together with use of oral contraceptive and hormone replacement therapy will be evaluated. Information on any alterations to or new medication prescribed by the participants’ doctors will also be collected by research staff via the monthly phone calls. Information recorded will include medication name, dose prescribed and daily quantity taken.

##### Health-related quality of life

Health-related quality of life will be assessed using the Short Form (36) version 2 questionnaire, which is a general measure of health status including eight scales: physical functioning, physical role functioning, bodily pain, general health perceptions, vitality, social role functioning, emotional role functioning and mental health [47]. This test yields a score from 0 to 100, where 0 represents the lowest and 100 represents the highest quality of life. Reliability scores of above 0.80 and empirical validities of 0.80 to 0.90 have been reported in the literature for both the physical and mental health measures [47].

##### Cognitive function

Cognitive function will be assessed using the CogState Brief Battery computerized tests [48], which provide sensitive and valid measurement of a range of different cognitive functions [49, 50]. The battery of tests that will be used for this study include: measures of executive function and spatial problem solving (Groton maze learning test), psychomotor function and speed of processing (detection task), visual attention (identification task), visual learning with a pattern separation model (one-card learning task) and working memory and attention (one-back task). Specific details about these five tests have been described previously [49–52]; all tests have been designed for repeated administration with minimal practice or learning effects.

##### Compliance

Compliance in taking the prescribed protein and vitamin D will be evaluated via self-completed compliance calendars and cross-referenced by counting remaining sachets and vitamin D capsules returned at each subsequent follow-up appointment. Compliance with the exercise programme will be evaluated via self-completed exercise cards, which will be initialled by the participant and viewed (signed off) by the trainer after each session and collected by the research staff from each health and fitness centre at approximately two-monthly intervals throughout the intervention.

##### Adverse events

Any adverse events associated with the exercise programme or supplements will be recorded by the research staff during the monthly phone calls to participants. For this study, an adverse event is defined as any health-related unfavourable or unintended medical occurrence (sign, symptom, syndrome, illness) that develops or worsens during the period of observation in the trial. All adverse events will be assessed for seriousness, causality and expectedness by the research staff and recorded and monitored during the trial.

### Sample size calculations

The sample size is based on the following power calculations from previously published studies of PRT in older adults (including those with type 2 diabetes) [10–12, 36, 38], and work of others that have assessed the independent or combined effects of protein, vitamin D and PRT on the outcome measures [16, 29, 53, 54]. It was estimated that 168 participants would provide 90% power (*P* <0.05 two-tailed test) to detect a 0.5% difference for the change in HbA_1c_ levels between the groups, assuming a conservative standard deviation of 1.1%. For insulin sensitivity, a sample size of 140 would be required to detect a 0.7 difference for the change in HOMA-2 insulin resistance between the groups at a power of 90%, assuming a conservative standard deviation of 1.2. To compensate for a projected 20% drop-out, a total of 202 participants will be recruited to the study and randomized 1:1 to the two groups (101 participants per group).

### Statistical analysis

The primary statistical analyses will be conducted on an intention-to-treat basis using STATA statistical software release 12.0 (STATA, College Station, TX, USA). Per-protocol analysis will also be performed by including all participants who are at least 80% compliant in completing the exercise (as measured by the number of exercise sessions attended) and in taking the drinks and supplements (as measured from the compliance calendar and pill count). Baseline characteristics between the groups will be compared by independent *t* tests for continuous variables and chi-square tests for categorical variables. Wherever possible, we will obtain endpoint measures from all withdrawals and include all randomized subjects in our final data analysis. All data will be checked for normality prior to analysis, and skewed data will be log transformed prior to analysis. Time, group and group-by-time interactions will be examined using generalized linear mixed models with random effects. Potential covariates to be included in the model will include: age, sex, race or ethnicity, changes in medication and change in habitual physical activity or diet. Multiple regression analysis will be used to investigate whether changes in lean tissue mass, muscle size, density and strength and a reduction in metabolic or inflammatory markers are predictive of any exercise-induced improvements in glycaemic control and insulin sensitivity. All data will be presented as mean ± standard deviation or 95% confidence intervals. The significance level will be set at *P* <0.05 or smaller if adjustments are made for multiple comparisons.

## Discussion

This study will be the first randomized controlled trial in older adults with type 2 diabetes to investigate whether increased dietary protein achieved through the ingestion of a whey-protein drink combined with vitamin D supplementation can enhance the effects of PRT on glycaemic control, muscle mass and cardiometabolic risk factors. This is important because most current lifestyle approaches for the treatment of type 2 diabetes focus on general physical activity (typically aerobic training) and calorie restriction to manage body weight. While such approaches have been shown to improve glycaemic control, blood pressure and lipid levels and reduce weight and fat mass, they are often associated with a loss in lean tissue (muscle) mass [6, 7]. Given that skeletal muscle is the largest mass of insulin-sensitive tissue and the predominant reservoir for glucose disposal, there is a need to develop, evaluate and disseminate approaches that are safe and effective for optimizing muscle mass as well as glycaemic control and other cardiometabolic related risk factors in people with this disease, particularly older adults with type 2 diabetes who experience an accelerated loss in muscle mass with age [55].

Progressive resistance training is one strategy that is now widely recommended for people with type 2 diabetes because of its beneficial effects on muscle mass, glycaemic control and other cardiometabolic risk factors that contribute to the development of diabetes and its complications. In non-diabetic adults, there is a growing body of evidence that post-exercise ingestion of a protein-rich source, such as whey-protein, can maximize the anabolic benefits of PRT on muscle [20–24]. Moreover, there is evidence that supplementation with vitamin D can have beneficial effects on muscle and measures of insulin sensitivity and secretion [25–28]. Therefore, this study will provide new information as to whether combining PRT with additional protein and vitamin D can promote a synergistic and incremental effect on glycaemic control, muscle mass and cardiometabolic risk factors compared with PRT alone in older adults with type 2 diabetes. If successful, this study will broaden the knowledge base and contribute to best practice guidelines on exercise and nutrition for the treatment of type 2 diabetes, along with the ongoing refinement of community-based initiatives for the management of this condition. In addition, the findings from this study will provide evidence to inform policy and translational activities of the existing community-based *Lift for Life®* programme, which is currently implemented throughout Australia.

## Trial status

Recruitment is currently underway and a number of participants have commenced the study.

## Authors’ original submitted files for images

Below are the links to the authors’ original submitted files for images. Authors’ original file for figure 1

## Abbreviations

25(OH)D: 25-hydroxyvitamin D
eGFR: estimated glomerular filtration rate
HbA_1c_: glycated haemoglobin
HOMA: homeostatic model assessment
HPLC: high performance liquid chromatography
IL-1β: interleukin-1β
IL-6: interleukin-6
IL-8: interleukin-8
IL-10: interleukin-10
PRT: progressive resistance training
TNF-α: tumor necrosis factor-α.
