# Supplementary figures and images for: The effects of progressive resistance training combined with a whey-protein drink and vitamin D supplementation on glycaemic control, body composition and cardiometabolic risk factors in older adults with type 2 diabetes: study protocol for a randomized controlled trial
Source: Trials. 2014 Nov 6;15:431. doi: 10.1186/1745-6215-15-431 (PMC4233106; doi:10.1186/1745-6215-15-431)

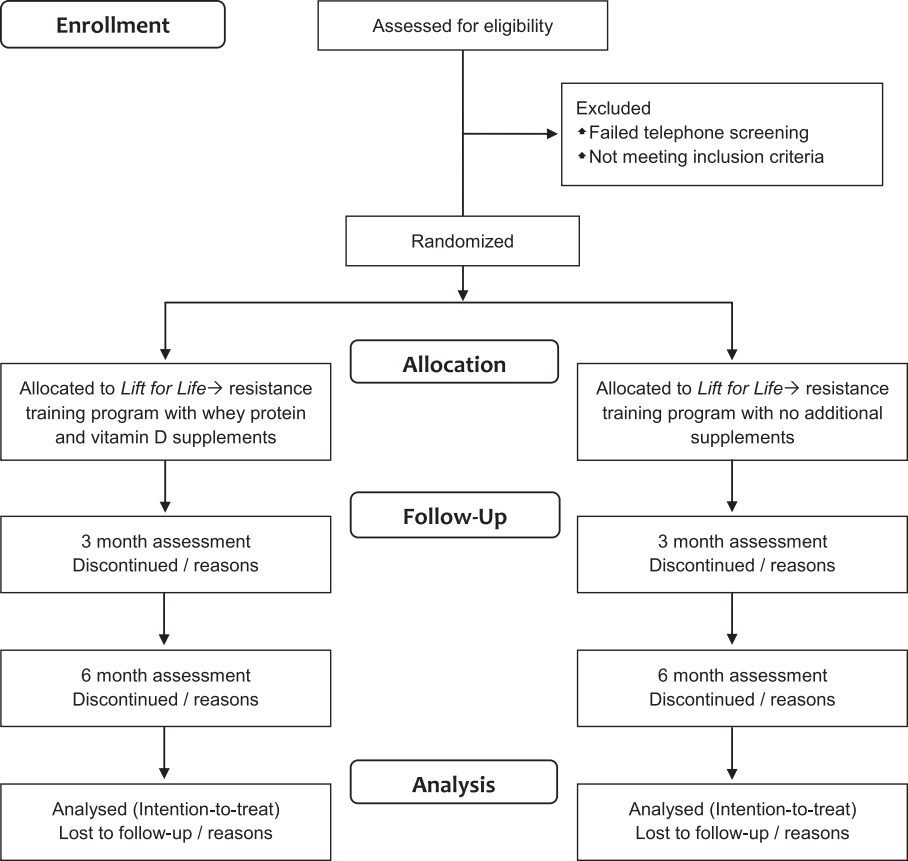

Supplement: Supplementary file 1 — Authors’ original file for figure 1 [file 13063_2014_2296_MOESM1_ESM.pdf]
